# An evaluation of *E. coli* in urinary tract infection in emergency department at KAMC in Riyadh, Saudi Arabia: retrospective study

**DOI:** 10.1186/s12941-018-0255-z

**Published:** 2018-02-09

**Authors:** Menyfah Q. Alanazi, Fulwah Y. Alqahtani, Fadilah S. Aleanizy

**Affiliations:** 1 Clinical Pharmacy Practice, Drug Policy and Economics Center, King Abdulaziz Medical City, Ministry of National Guard, Health Affairs, Riyadh, 22490 Saudi Arabia; 2 Department of PharmaceUTIscs, College of Pharmacy, King Saud University, Riyadh, 22452 Saudi Arabia

**Keywords:** Antibiotics resistance, *E. coli*, Empirical therapy, UTIS

## Abstract

**Background:**

Urinary tract infection (UTIS) is a common infectious disease in which level of antimicrobial resistance are alarming worldwide. Therefore, this study aims to describe the prevalence and the resistance pattern of the main bacteria responsible for UTIS *Escherichia coli* (*E. coli*).

**Methods:**

Retrospective chart review for patients admitted to emergency department and diagnosed with UTIS at KAMC, in Riyadh, Saudi Arabia between January to March 2008 was performed. Antimicrobial susceptibility to ampicillin, augmentin (amoxicillin/clavulanate), cefazolin, co-trimoxazole (sulfamethoxazole/trimethoprim), ciprofloxacin, and nitrofurantoin, and cefpodoxime was determined for 101 *E. coli* urinary isolates.

**Results:**

*Escherichia coli* was the most prevalent pathogen contributing to UTIS representing 93.55, 60.24, and 45.83% of all pathogen isolated from urine culture of pediatric, adult, and elderly, respectively. High rates of resistance to ampicillin (82.76, 58, and 63.64%) and co-trimoxazole (51.72, 42, and 59.09%), among *E. coli* isolated from pediatric, adult and elderly respectively. Nitrofurantoin was the most active agent, followed by ciprofloxacin, augmentin and cefazolin. 22.77% of *E. coli* isolates exhibited multiple drug resistance (MDR). Among 66 and 49 isolates resistant to ampicillin and co-trimoxazole, respectively, 34.84 and 42.85% were MDR. In contrast, all isolates resistant to augmentin and nitrofurantoin were MRD, while 72.7 and 82.4% of isolates resistant to ciprofloxacin and cefazolin were MDR.

**Conclusions:**

High resistance was observed to ampicillin and co-trimoxazole which commonly used as empirical treatments for UTIS, limiting their clinical use. This necessitates continuous surveillance for resistance pattern of uropathogens against antibiotics.

## Background

Urinary tract infections (UTIs) are among the most common types of bacterial infections occurring both in the community and hospital settings [1, 2]. There are two types of UTIs: hospital associated urinary tract infection (HAUTIs), and community-associated urinary tract infection (CAUTIs) [3]. Women are the predominant group of patients with CAUTIs [1, 2]. UTIs was estimated to represent 100,000 hospitalizations, 7 million visits and 1 million admissions to emergency services in USA [4, 5]. The economic and public health burdens of UTIs is substantial and markedly affect the quality of life of infected patients [6]. In Saudi Arabia, UTIs were reported to be the second leading cause of infections predominately in women at ED [7].

The majority of UTIs are caused by *E. coli* bacteria, followed by *Proteus* spp., *Staphylococcus saprophyticus*, *Klebsiella* spp. and other *Enterobacteriaceae* [8, 9]. However, among bacteria causing UTIS, *E. coli* is considered as the most predominant cause of both community and nosocomial UTIs. Antibiotics commonly recommended for treatment of UTIs include co-trimoxazole (trimethoprim/sulfamethoxazole), nitrofurantoin, ciprofloxacin and ampicillin [3, 10]. However, there is global increase in antibiotic resistance among urinary tract pathogens, including resistance pattern observed in Saudi Arabia [11–14], which limit treatment options.

Evidence suggest significant relationship between extensive use of antimicrobial and antimicrobial resistance [10, 11]. Therefore, appropriate antibiotic prescription and usage will reduce the disease burden of UTIs and hence lower its complications and costs [10, 15]. For this reason, surveillance of antibiotics resistance is crucial to determine the pattern of antimicrobial resistance and consequently guide the selection of empirical therapy. Therefore, this study aims to determine the current prevalence and susceptibility to ampicillin, augmentin (amoxicillin/clavulanate), co-trimoxazole (trimethoprim/sulfamethoxazole), nitrofurantoin, cefazolin, and ciprofloxacin amongst all *E. coli* isolated from patients with UTIs. Associations between patient’s demographic parameters and multidrug resistance *E. coli* isolates were also investigated.

## Methods

### Study setting

This study was conducted in the emergency department (ED) of King Abdulaziz Medical City (KAMC), which is a tertiary care hospital in Riyadh, the capital of Saudi Arabia. The ED at KAMC is composed of Urgi Center, Pediatric Emergency Units, Adult Emergency unit, Observation Unit, and Critical Care unit.

### Study design

Retrospective cohort study of physician medication prescription over a period of 3 months from January to March 2008 in ED at KAMC, Riyadh, Saudi Arabia.

### Study population

Charts of 101 patients diagnosed with UTIs due to *E. coli* were reviewed. Samples of those patients showed significant growth, bacteria growing > 10^5^ colony-forming units (CFU/mL) with a single type of bacteria from a properly collected midstream “clean catch” urine sample, were considered as UTIs and processed further for identification and susceptibility testing.

### Antibiotic sensitivity testing

The sensitivity and resistance of *E. coli* isolates against six antimicrobial agents including ampicillin, augmentin, co-trimoxazole (trimethoprim/sulphamethoxazole), ciprofloxacin, nitrofurantoin, and cefazolin were determined using VITEK test method which measure minimum inhibitory concentration (MIC). Susceptibility results are interpreted according to the Clinical Laboratory Standards Institute (CLSI) guidelines. Then association between resistance of *E. coli* to one or more of three antibiotics (ampicillin, co-trimoxazole and ciprofloxacin) were studied and classified to SSR (sensitive to ampicillin, sensitive co-trimoxazole, and resistance ciprofloxacin); RRR (resistance ampicillin, resistance co-trimoxazole and resistance ciprofloxacin); SSS (sensitive ampicillin, sensitive co-trimoxazole and Sensitive ciprofloxacin); RRS (resistance ampicillin, resistance co-trimoxazole and sensitive ciprofloxacin); SRS (sensitive ampicillin, resistance co-trimoxazole and sensitive ciprofloxacin), and SRR (sensitive ampicillin, resistance co-trimoxazole and resistance ciprofloxacin). Multiple resistance which defined as multidrug resistance (MDR) was defined as resistance to three or more antimicrobials. The population was classified as pediatric, adolescent and adult, and elderly. Pediatric stratified into three groups: less than 2 years, from 2 years to 6 years and between 7 and 12 years. The age category between 13–17 and 18–64 years were defined as adults, while those age 65 years or over were defined as elderly.

### Measurement

Data for the period of January 1, 2008, through March 30, were obtained from the prescription and Quadra Med system. All prescriptions with UTIs diagnosis were collected and reviewed for the demographic characteristic, the name of antibiotics and microbiology data. Microbiology data include name of the uropathogen which isolated from urine culture and cause UTIs, sensitivity, and resistance of *E. coli* for six antimicrobials agents as prescribed above.

### Statistical analysis

Antimicrobial susceptibility or resistant rates were calculated as the number of susceptible or resistant organisms divided by the total number of tested organisms, for a given antibiotic, and a given organism. Statistical analyses were performed using the Statistical Package for the Social Sciences (SPSS version 16.0).

### Ethical approval

This study approved by the research committee at King Abdullah International Medical Research Center, Riyadh, Saudi Arabia.

## Results

During the study period from January to March 2008, a total 565 urinary cultures were collected. Of a total isolate, positive cultures were representing 28.67% of all cultures collected. Gram-negative organisms totaled 149 (91.98%), Gram-positive organisms constituted 11 (6.79%) and fungal 2 (1.23%) of all isolates. There are 56.14% of all pediatric visits requested culture. 35.67% of all adult visits and 39.75% of all elderly. In pediatric, 32.29% of culture was positive, 62.50% was negative, and 5.21% of the result was mixed of normal flora, while in the adult the result of urine culture showed 68.62% negative, 24.34% positive and 7.05% was mixed. In elderly, the result of culture was 57.81% negative, 37.50% positive and 5.21% mixed of normal flora.

The most common uropathogen isolated from urine culture was *E. coli*, which represent 93.55% of all pathogen that isolated from pediatric urine culture, 60.24% of all pathogen which isolated from adult urine culture and 45.83% of all pathogen that isolated from elderly urine culture (Table 1). Extended Spectrum Beta-Lactamases enzymes (ESBL) producing *E. coli* detected in 8.33, 4.82 and 3.23% of uropathogens isolated from elderly, adult and pediatric respectively. Other organisms, in elderly, were *Enterobacter* species (10.42%), *Klebsiella* species (10.42%), and *Acinetobacter* species (6.25%), and *Pseudomonas aeruginosa* (4.14%). Other organisms were caused UTIs in adult were *Klebsiella* species (10.42%) *Pseudomonas aeruginosa* (8.43%), and *Streptococcus* species (8.43%) (Table 1).

**Table 1:** Distribution of uropathogens that cause urinary tract infection according age category in period from January to March 2008

Uropathogens	Frequency among patients					
	Elderly (n = 48)		Adult (n = 83)		Pediatric (n = 31)	
	n	%	n	%	n	%
*Escherichia coli*	22	45.83	50	60.24	29	93.55
*Enterobacter* species	5	10.42	4	4.82	0	0
*Klebsiella* species	5	10.42	7	8.43	0	0
Extended spectrum B lactamase (ESBL)	4	8.33	4	4.82	1	3.23
*Acinetobacter* species	3	6.25	1	1.20	0	0
*Group B sterptococcus*	2	4.17	7	8.43	0	0
*Pseudomonas aeruginosa*	2	4.17	7	8.43	0	0
*Candida* species	1	2.08	1	1.20	0	0
*Morganella morganii*	1	2.08	0	0.00	0	0
*Proteus merabilis*	1	2.08	1	1.20	1	3.23
*Serratia marcescens*	1	2.08	0	0.00	0	0
*Staphylococcus aureus*	1	2.08	0	0.00	0	0
*Methicillin resistance Staphylococcus aureus* (MRSA)	0	0	1	1.20	0	0

The overall rate of resistance for 101 *E. coli* isolates analyzed was provided in Table 2. Of the agents tested nitrofurantoin, ciprofloxacin and augmentin were the lowest rates of resistance in three age categories. Nitrofurantoin was the lowest rate of resistance among three age categories representing (0, 2, 9%) followed by ciprofloxacin representing (6.90, 8, 27.27%), and augmentin representing (10.34, 10, 27.27%) in pediatric, adult and elderly respectively. Ampicillin demonstrated the highest rate of resistance (82.76, 58, and 63.64%) in pediatric, adult and elderly respectively. The rate of co-trimoxazole resistance among the *E. coli* isolates was (51.72, 42%), and 59.09% in pediatric, adult and elderly respectively (Table 2).

**Table 2:** Antimicrobial susceptibility result for 101 *E. coli* urinary tract isolates in study period from January to March 2008

Antibiotic name	Number of isolates n (%) classified as					
	Pediatric (n = 29 isolates)		Adult (n = 50 isolates)		Elderly (n = 22 isolates)	
	Sensitive	Resistance	Sensitive	Resistance	Sensitive	Resistance
Ampicillin	5 (17.24%)	24 (82.76%)	21 (42%)	29 (58%)	8 (36.36%)	14 (63.64%)
Augmentin	26 (89.66%)	3 (10.34%)	45 (90%)	5 (10%)	16 (72.73%)	6 (27.27%)
Co-trimoxazole	14 (48.28%)	15 (51.72%)	29 (58%)	21 (42%)	9 (40.91%)	13 (59.09%)
Ciprofloxacin	27 (93.1%)	1 (3.44%)	46 (92%)	4 (8%)	16 (72.73%)	6 (27.27%)
Nitrofurantoin	29 (100%)	0 (0.00%)	49 (98%)	1 (2%)	20 (90.91%)	2 (9.09%)
Cefazolin	25 (86.21%)	4 (13.79%)	43 (86%)	7 (14%)	15 (68.18%)	7 (31.82%)

Co-trimoxazole (sulfamethoxazole/trimethoprim)

Antimicrobial resistance to individual antibiotics and the percentage of isolates demonstrating MDR phenotype were stratified by patient demographic characteristic and summarized in (Table 3). Nitrofurantoin resistance was approximately three times as common among *E. coli* isolates from males (7.14%) more than females (2.3%) and was highest (9.09%) among patients > 65 years old. Augmentin resistance was approximately comparable among *E. coli* isolates from male (14.29%) and female (13.79%) and the highest (27.27, and 15.78%) among patients more than 65 years old and equal or less than 6 years old respectively. Higher rates of ciprofloxacin resistance (27.27%) among patients > 65 years was demonstrated (Table 3). There was no resistance against augmentin and cefazolin among *E. coli* isolated from patients aged between 7 years to 17 years old. The rate of resistance of *E. coli* to co-trimoxazole was high in patients < 2 years old and > 65 years old representing 85.71% and 95.09%, respectively. Trend toward higher rates of MDR *E. coli* were demonstrated among males’ patients (28.57%). By age category, the prevalence of MDR was higher in elderly 50% (n = 11 of 22), followed by adult 18% (n = 9 of 50), and pediatric 10.34% (n = 3 of 29) (Table 3).

**Table 3:** Patients demographic characteristic for 101 *E. coli* urinary tract isolates during period from January to March 2008

	Total no isolates	Susceptibility to all antibiotic	Resistance to one or more	n (%) isolate resistance to						
				Ampicillin	Augmentin	Co-trimoxazole	Ciprofloxacin	Nitofurantoin	Cefazolin	% MDR
Gender										
Male	14 (13.86%)	4 (28.57%)	10 (71.42%)	9 (64.29%)	2 (14.29%)	6 (42.86%)	2 (14.29%)	1 (7.14%)	2 (14.29%)	4 (28.57%)
Female	87 (86.14%)	28 (32.18%)	59 (67.82%)	57 (65.52%)	12 (13.79%)	43 (49.43%)	9 (10.34%)	2 (2.3%)	15 (17.24%)	19 (21.84%)
Total	101	32 (31.68%)	69 (68.31%)	66 (65.34%)	14 (13.9%)	49 (39.60%)	11 (10.90%)	3 (3%)	17 (16.83%)	23 (22.77%)
Category of age										
< 2 years	7 (6.93%)	1 (14.29%)	6 (85.71%)	6 (85.71%)	1 (14.29%)	6 (85.71%)	0 (0)	0 (0)	1 (14.29%)	1 (14.29%)
2 to 6 years	12 (11.88%)	3 (25%)	9 (75%)	9 (75%)	2 (16.67%)	6 (50%)	0 (0)	0 (0)	2 (16.67%)	2 (16.67%)
7 to 12 years	10 (10%)	2 (20%)	8 (80%)	8 (80%)	0	3 (30%)	1 (10%)	0 (0)	0 (0)	0 (0)
13 to 17 years	2 (2%)	1 (50%)	1 (50%)	1 (50%)	0	1 (50%)	0 (0)	0 (0)	0 (0)	0 (0)
18 to 64 years	48 (47.52%)	18 (37.5%)	30 (62.50%)	28 (58.33%)	5 (10.42%)	20 (41.67%)	4 (8.33%)	1 (2.08%)	7 (14.58%)	9 (18.75%)
65 years or more	22 (21.78%)	7 (31.82%)	15 (68.18%)	14 (63.64%)	6 (27.27%)	13 (59.09%)	6 (27.27%)	2 (9.09%)	7 (31.82%)	11 (50%)
Total	101	32	69	66	14	49	11	3	17	23

Among the 101 *E. coli* isolates that were tested against all six antimicrobials, 31.68% found to be susceptible (Table 4). The majority (68.31%) was resistant to one or more antimicrobial and 15.84% were resistant to a single agent, predominantly ampicillin, and 29.7% were resistant to two antimicrobials. MDR isolates accounted for 22.77% (n = 23) of the 101 isolates. The majority of MDR isolates (n = 13; 56.52%) were resistant to three antimicrobial, and these accounted for 12.87% of all isolates. Isolates were also identified to be resistant to four agents were (n = 8; 34, 78% of MDR isolates; 7.92% of all isolates) and all five antimicrobials were (n = 2; 8, 69% of MDR isolates; 1.98% of all isolates) (Table 4). Resistant to ampicillin represent 34.84% (n = 23 of 23) of the MDR isolates. Among 66 and 49 isolates resistant to ampicillin and co-trimoxazole, respectively, 34.84 and 42.85% were MDR (Table 4). In contrast, all isolates resistant to augmentin and nitrofurantoin were MRD, while 72.7 and 82.4% of isolates resistant to ciprofloxacin and cefazolin were MDR.

**Table 4:** Resistance to one or more antimicrobials among 101 *E. coli* urinary tract isolates tests against all six antimicrobials in study period

No of agents which isolates were resistance	N, % of isolates	n (%) isolates resistant to					
		Ampicillin	Augmentin	Co-trimoxazole	Ciprofloxacin	Nitrofurantoin	Cefazolin
0	32 (31.68%)						
1	16 (15.84%)	14 (87.50%)	0	2 (12.50%)	0	0	1 (6.25%)
2	30 (29.70%)	29 (96.67%)	0	26 (86.67%)	3 (10%)	0	2 (6.67%)
3	13 (12.87%)	13 (100%)	5 (38.46%)	12 (92.31%)	5 (38.46%)	0	5 (38.46%)
4	8 (7.92%)	8 (100%)	7 (87.5%)	7 (87.50%)	2 (25%)	2 (25%)	7 (87.50%)
5	2 (1.98%)	2 (100%)	2 (100%)	2 (100%)	1 (50%)	1 (50%)	2 (100%)
Total	101	66	14	49	11	3	17
MDR	23	23	14	21	8	3	14
% MDR	77.22%	34.84%	100%	42.85%	72.72%	100%	82.35%

The analysis of co-resistance between ampicillin, co-trimoxazole and ciprofloxacin were summarized in Table 5. The correlation between ampicillin and co-trimoxazole were 51.72, 34 and 27.27% in pediatric, adult and elderly respectively. *E. coli* were resistant to ampicillin, ciprofloxacin, and co-trimoxazole in adult and elderly were 4 and 27.27% respectively. In adult, the correlation between resistance of ciprofloxacin and co-trimoxazole was 2% (Table 5).

**Table 5:** Resistance profile to the three antibiotics (ampicillin, ciprofloxacin and co-trimoxazole) among 101 isolates of *E. coli*

Antibiotype	Ampicillin	Co-trimoxazole	Ciprofloxacin	Pediatric n (%)	Adult n (%)	Elderly n (%)	Total n (%)
A	S	S	S	6 (20.69%)	19 (38%)	7 (31.82%)	32 (31.68%)
B	R	R	S	15 (51.72%)	17 (34%)	6 (27.27%)	38 (37.62%)
C	R	R	R	0	2 (4%)	6 (27.27%)	8 (7.92%)
D	R	S	R	1 (3.45%)	1 (2%)	0	2 (1.98%)
E	R	S	S	7 (24.14%)	9 (18%)	2 (9.09%)	18 (17.82%)
F	S	R	R	0	1 (2)	0	1 (0.99%)
G	S	R	S	0	1 (2)	1 (4.55%)	2 (1.98%)
Total				29	50	22	101

*S* susceptible, *R* resistance

## Discussion

Global spread of antibiotics resistance among uropathogens causing UTIs is alarming. This study reports the etiology of UTIs and antimicrobial susceptibility of uropathogens at KAMC in Riyadh, a capital city of Saudi Arabia. Similar to previous studies that conducted in Saudi Arabia, *E. coli* remains the majority of pathogen which isolated from urine culture in pediatric, adult, and elderly [11–14, 16], and other countries [17–19].

In this study, the resistance of *E. coli* to ampicillin was the highest, followed by co-trimoxazole. The observed resistance pattern of *E. coli* to ampicillin and co-trimoxazole are in agreement with the findings of previous studies carried out at different provinces of Saudi Arabia [11–13, 16]. Other study taken place in USA showed that that MDR *E. coli* exhibited 97.8% resistance to Ampicillin, 92.8% to trimethoprim–sulfamethoxazole, and 38.8% to ciprofloxacin [20]. In UK, high rates of resistance to ampicillin (55%) and trimethoprim (40%) were observed in *E. coli* isolates [18]. These results suggest the prevalence of ampicillin and trimethoprim/sulfamethoxazole resistance among urinary tract isolates and are consistent with finding of our study. *E. coli* showed the highest sensitivity to nitrofurantoin in the current study, which are in line with those of previous studies in Saudi Arabia [12, 14], and other countries [21, 22]. These data further support recommendations made in previous studies, in which, nitrofurantoin may be more effective than co-trimoxazole or amoxicillin in the empiric treatment of UTIs [21, 23].

The current data showed a high percentage of correlation between co-trimoxazole and ampicillin resistance among *E. coli* isolates (37.62%). Previous data from Canadian study revealed limited activity of ampicillin (79.6% resistance) against co-trimoxazole-resistant *E. coli* isolates from UTIs patients [24]. In the same study, the rates of co-trimoxazole resistance for ampicillin-resistant *E. coli* was (35.7% resistance) [24], corroborating finding of our study. In addition, high ciprofloxacin resistant (100%) was found among co-trimoxazole resistance isolates [24]. This finding is contrary to current study results as there is no evidence of high prevalence in ciprofloxacin resistant among ampicillin and/or co-trimoxazole resistant *E. coli* isolates from UTIs.

The current study found that 22.77% of *E. coli* isolates were MDR, which is higher than that observed in ED of previous study [25]. MDR prevalence was higher in *E. coli* isolates resistant to augmentin, nitrofurantoin, followed by cefazolin and ciprofloxacin, then co-trimoxazole and ampicillin.

Despite the lowest rate of nitrofurantoin resistance (3%) when compared to other antibiotics used in the current study (only 3 of 101), these isolates were MDR. Similarly, 100% of isolates resistant to augmentin were also MDR. However, in our study we described MDR phenotypes for ampicillin, co-trimoxazole, and ciprofloxacin but not for other antibiotics. Therefore, we cannot conclude the correction between MDR with specific antibiotic.

According to patient age, the prevalence of resistance to ampicillin or co-trimoxazole was higher among isolates from patients ≤ 12 years and ≤ 2 years, respectively. This pattern seemed relatively consistent with findings of previous study which revealed higher level of resistance among isolates from patients ≤ 17 years old than among older patients [22]. For augmentin, cefazolin, ciprofloxacin, and nitrofurantoin, the prevalence of resistant isolates were higher among patients aged ≥ 65 years. This match results observed in earlier studies that found ciprofloxacin resistance was highest among patients older than 65 years (7.1%) [22]. However, nitrofurantoin resistance pattern in our study is contrary to that described by Sham et al. study, in which resistance prevalence of nitrofurantoin resistance was consistent irrespective of patient age [22].

The present study has some limitations. First, the study was only conducted only in ED setting, which not representing other health care settings in KAMC. Thus, the findings cannot be generalized. Second, the study period was short (3 months) and small sample size. Third, there was no follow-up study to investigate the consequences of observed resistance. Therefore, large-scale prospective studies are recommended to determine the extent and outcomes of resistance and MDR in UTIs at KAMC in Saudi Arabia.

## Conclusions

Increased resistance of urinary tract *E. coli* isolates to both ampicillin and co-trimoxazole was demonstrated, suggesting revaluation empirical therapies for the treatment of UTIs in ED setting. This continuous evolvement in antimicrobial resistance pattern necessitates national surveillance studies to monitor and ensure safe and effective empiric therapy.

## Abbreviations

KAMC: King Abdulaziz Medical City
KAIMRC: King Abdullah International Medical Research Center, augmentin, (amoxicillin/clavulanate
ED: emergency department, co-trimoxazole, (sulfamethoxazole/trimethoprim)
UTIs: urinary tract infections
MDR: multiple drug resistance
MRSA: methicillin resistance *S. aureus*
